# Inhibition of Autophagy in Heat-Stressed Sperm of Adult Mice: A Possible Role of Catsper1, 2 Channel Proteins

**DOI:** 10.1155/2023/6890815

**Published:** 2023-10-09

**Authors:** Malihe Soltani, Majid Rahmati, Mohammad Reza Nikravesh, Shahin Saeedi Nejat, Mahdi Jalali

**Affiliations:** ^1^Department of Anatomy, Faculty of Medicine, Gonabad University of Medical Sciences, Gonabad, IR, Iran; ^2^Department of Medical Biotechnology, School of Medicine, Shahroud University of Medical Sciences, Shahroud, IR, Iran; ^3^Departments of Anatomy and Cell Biology, Faculty of Medicine, Mashhad University of Medical Sciences, Mashhad, IR, Iran; ^4^Schools of Persian and Complementary Medicine, Mashhad University of Medical Sciences, Mashhad, IR, Iran

## Abstract

**Objective:**

Various phenomena guarantee gamete maturation and formation at all stages of evolution, one of which is autophagy playing a critical role in the final morphology of gametes, particularly sperms. Autophagy is influenced by oxidative stress, disturbances of calcium homeostasis, and hyperthermia conditions. The current study aimed to assess the autophagy-related proteins along with the activity of sperm calcium channel (CatSper) proteins following the induction of heat stress (HS).

**Methods:**

The study sample includes two groups of adult mice: sham and HS groups. In the HS group, the right testis was transferred to the abdominal cavity for 120 hours and then returned to the scrotum where it remained for 7 days. After 7 days, the testis and epididymis were removed to conduct real-time, immunohistochemical studies, sperm parameter evaluation, and seminiferous tubule assessment. In this study, the expression and distribution of autophagy proteins were measured. Plus, CatSper1 and CatSper2 were evaluated as proteins of calcium channels.

**Results:**

The results of the present study demonstrated that the expression intensity of autophagy indices in seminiferous tubules decreased significantly after HS induction, which was associated with a decrease in the distribution of CatSper proteins in the sperms. HS led to morphological changes in sperm, reduced motility and viability of sperm, and decreased spermatogenesis indices.

**Conclusion:**

In this study, following heat stress, the decrease in CatSper protein distribution may lead to the structural disorder of CatSper channels, which could strongly affect autophagic activity. Also, disruption of spermatogenesis and sperm parameters may be the consequence of decreased autophagy activity.

## 1. Introduction

Autophagy is a hemostatic and fully protected phenomenon throughout growth and survival. The autophagy lysosomal pathway (ALP) authorizes the removal and replacement of proteins and nonfunctional organs by recycling compounds and renovating them into functional macromolecules [1]. In sperms, autophagy ensures structural and morphological modifications at the spermiogenesis stage [2, 3]. During episodes of food deprivation in sperm, calcium activates autophagy through a signaling pathway called Ca MKKB/AMPK/mTOR, so impaired calcium homeostasis is associated with the inhibition of autophagy [4, 5]. The most vital channel for calcium transportation into the sperm cytoplasm is the sperm-specific cation channel or CatSpers. CatSper1 and 2, as genes regulating spermatogenesis, play a critical role in controlling sperm motility and are expressed exclusively in the testis [6]. It has also been indicated that in various cells of the body, including neurons and heart cells, damage to calcium channels prevents an increase in cytoplasmic calcium concentration and leads to autophagy inhibition [4, 7]. Therefore, it is not unreasonable to expect that in sperm, with damage to CatSper channels and changes in calcium homeostasis, sperm parameters and autophagy activity change. Various stressful situations can lead to inhibited function or destruction of calcium channels, such as heat stress (HS) situation [8]. HS occurs when the temperature exceeds a physiological range in the testis in conditions such as cryptorchidism in adolescents or retractile testis (RT) in adults [9]. In the present study, the RT model was utilized to induce HS. RT is commonly known as a variant of normal testicles that have retracted to the suprascrotal position during infancy, adolescence, or puberty for a variety of reasons. Its prevalence was 4.5 to 13 cases per 1000 individuals [10–12]. Among testicular tissue cells, spermatids and spermatocytes are the most sensitive to heat stress. Several studies have revealed that HS is associated with significant morphological abnormalities and impaired sperm motility [13]. In the present study, it is hypothesized that HS might be able to affect the distribution of autophagy proteins including LC3*β* (microtubule-associated protein 1 Light Chain 3-phospholipid conjugates) and Beclin-1 (autophagy-relatedbcl2-interacting Atg6 homolog) by disrupting CatSper1 and CatSper2 calcium proteins. Consequently, it is expected that abnormalities and damage to sperm parameters ensue following autophagy inhibition.

## 2. Materials and Methods

### 2.1. Animals

In this experimental study, 16 male Balb/C mice with approximate weights of 20 to 25 gr and about 9-weeks-old were prepared from the animal house of Mashhad University of Medical Sciences. They had been kept in the standard conditions of the animal house, including a 12/12 h light/dark cycle, room temperature at 23 ± 2°C, and 60%–70% humidity. Mice in all groups had free access to food and water. Ethical considerations were taken into account according to the principles of the Vice Chancellor for Research and Technology of Mashhad University of Medical Sciences (No. 971786). Mice were randomly divided into two experimental groups:

  Group 1: incision and suturing on the scrotum wall without induction of HS (sham).
  Group 2: induction of HS for 120 hours in the right testis (HS).

### 2.2. Surgical Procedure

In the HS induction group, the mice were anesthetized with 50 mg/kg ketamine and 10 mg/kg xylazine. Afterward, through a longitudinal incision to the right scrotum, the testis appeared. Then the testicle and spermatic cord were separated from the surrounding fascia then transferred to the abdomen. There, the tunica vaginalis of the testis was fixed to the abdominal wall with a single suture (6–0 silk). Finally, the scrotum was sutured with 5–0 silk. Once the HS induction period (120 h) was completed, the testes were returned to the scrotum [9]. To prevent retrieval to the abdomen, the testes were fixed in the scrotum with a suture. After 7 days, the mice were anesthetized using ketamine-xylazine. In this study, all surgical procedures and anesthesia materials were performed in the sham group just like the HS group, except that the testicles were not placed in the abdominal cavity. For histological and immunohistochemical studies, the right testis was removed and fixed in formalin. Also, the tail of epididymis was separated and cut into two pieces. One of the pieces was utilized for real-time PCR molecular studies, and the other one was used to assess sperm parameters [14].

### 2.3. Testicular Histology and Sperm Parameter Analysis

Sperm parameters were studied morphologically in the head and the tail. Adult sperm tails were evaluated based on frequency in the sham group, i.e., the average length of most sperm tails in the sham group was considered as a longitudinal pattern to be compared with other sperms. At this stage, a sickle-shaped head and a normal tail were considered control patterns [15]. To evaluate sperm viability after RT induction, eosin Y staining was used. In this staining method, the sperm sample was mixed with eosin Y at a ratio of 4 : 1. After a few minutes, some of the above mixtures were spread on the Neubauer slide. Then, 200 sperms were evaluated in ten random fields using a light microscope (Olympus BX51, Japan). Based on the penetration of eosin Y into the cell membrane of sperms, red sperms were reported as dead sperms and colorless sperms as viable sperms.

In the present study, the spermatogenesis index of seminiferous tubules was measured according to Johnson's scoring system. This scoring system is based on the determination of tissue characteristics in pathological lesions. Johnson's scoring system is based on the determination of tissue characteristics in pathological lesions. To do so, seminiferous tubules were examined in each round cross section, and a score of 1–10 was assigned to each cross section [16]. The seminiferous tubes were selected randomly, and 30 round tubes were selected from each slide to conform to the histology guide. Furthermore, the diameter of seminiferous tubules and the height of their epithelium were calculated at stages 7 and 8. To calculate the diameter of seminiferous tubules, round cross sections were randomly selected. Then, the diameter of each tubule was calculated from the base membrane of one side to the other side of the tube by using ImageJ software. The height of the seminiferous epithelium in micrometers was also calculated at 400x magnification using ImageJ software.

### 2.4. Immunohistochemical Analysis

In this technique, after the testicular tissue was fixed in 10% formalin, tissue processing was performed. In the next steps, the peroxidase activity of the sample was prevented. Also, in the presence of 10% goat serum solution, the activity of nonspecific antigens was inhibited. Samples were covered with primary and secondary antibodies, respectively. After that, when samples were exposed to diaminobenzidine (DAB) solution, reactions were detected with secondary antibodies in brown color. After dehydrating and fixing the slides, the stained slides were observed and evaluated using a light microscope (Olympus BX51, Japan), and the intensity of protein expression was quantified by ImageJ software [14]. Beclin-1 (E-8): sc-48341, Santa Cruz, USA, LC3*β* (G-9): sc-incubated. 376404, Santa Cruz, USA, CatSper1: D-17:sc21180, and CatSper2: L-17:sc83119. Also, the secondary antibodies used were as follows: m-IgG*κ* BP-HRP: sc-516102, Santa Cruz, USA and donkey polyclonal- Anti goat: Bio-Rad AbD Serotec, 7061. In the present study, IHC analysis of LC3*β* proteins and Beclin-1 was enacted on testicular tissue sections and CatSper1 and CatSper2 on sperm smears. At stages 7 and 8 of spermatogenesis, the distribution of LC3*β* and Beclin-1 proteins of testicular seminiferous tubules was assessed. Brown spots indicating the presence of autophagosomes were detected.

### 2.5. Real-Time Analysis

To perform the real-time technique, the epididymis was homogenized using a homogenizer. The total sperm RNA was extracted according to the protocols of the kit manufacturer. Then, the purity of RNA was determined on agarose gel through electrophoresis. RNA reverse transcription was performed by the cDNA synthesis kit according to the kit manufacturer's protocols. Furthermore, RT-PCR was performed within the ABI PRISM®48 device. The *GAPDH* gene was used as an internal control gene. For the relative measurement of gene expression changes, the 2^−ΔΔCT^ method was utilized [17]. The sequence of oligonucleotide primers utilized in RT-PCR is as follows: *GAPDH* (forward primer: *CCAATGTATCCGTTGTGGATC*/reverse primer: *ATTGTCATACCAGGAAATGAGC*),*Beclin-1* (forward primer: *AGGCTGAGGCGGAGAGATTG*/reverse primer: *TGTGGAAGGTGGCATTGAAGAC*), and *MAPLC3β* (forward primer: *CACTGCTCTGTCTTGTGTAGGTTG*/reverse primer: *TCGTTGTGCCTTTATTAGTGCATC*).

### 2.6. Statistical Methods of Data Analysis

Statistical analysis was accomplished within SPSS 20 software (IBM; USA). All data were presented in *M* ± SE format. A one-way ANOVA test followed by Tukey's range test was conducted to compare immunohistochemical values. An independent *T*-test was executed and analyzed to compare the data of the real-time PCR technique. *p* ≤ 0.05 was considered statistically significant.

## 3. Result

### 3.1. Evaluation of Seminiferous Histological Marker and Sperm Parameters

#### 3.1.1. The Spermatogenesis Index of Seminiferous Tubules

**Figure 1:**
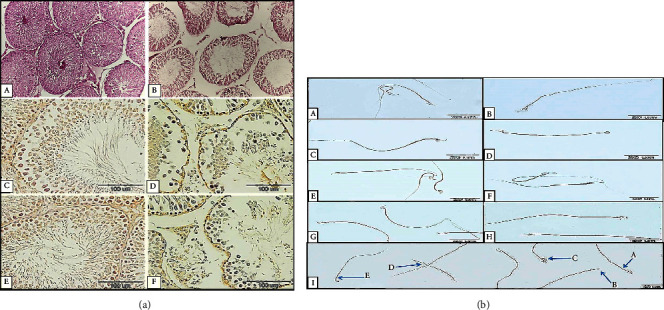
(a) Testicular histology and immunohistochemical findings in sham and HS groups. Evaluation of the seminiferous histological marker (testicular modified Johnsen score, seminiferous tubule diameter, and epithelium height) in sham (A) and HS (B) groups, H&E ×400. Evaluation of autophagy marker (LC3*β*, Beclin-1) in seminiferous tubule. Photomicrographs show immunoreactivity of LC3*β* ((C) sham and (D) HS groups) and Beclin-1 ((E) sham and (F) HS groups). (b) Evaluation of sperm autophagy marker (LC3*β*, Beclin-1) and CatSper1, 2 proteins. The positive immunoreactivity of these proteins is shown in brown color in the sperm body. Photomicrographs show immunoreactivity of LC3*β* ((A) sham and (B) HS groups), Beclin-1 ((C) sham and (D) HS groups), CatSper1 ((E) sham and (F) HS groups), and CatSper2 ((G) sham and (H) HS groups). (I) Abnormal sperm in HS group (A: normal sperm; B: headless sperm; C and E: sperm with an abnormal head; D: short tail sperm).

To determine the spermatogenesis index of seminiferous tubules, Johnson's scoring system was utilized at stages 7 and 8. The result obtained concerning the mean score in the sham group was equal to 9.54, and statistical evaluations revealed that there was a statistically significant decrease in the HS group compared with the sham group (*p* = 0.022) (Table 1) (Figure 1(a)).

#### 3.1.2. Seminiferous Tubule Diameter and Epithelium Height

In the present study, the diameter of seminiferous tubules decreased in the HS group as opposed to the sham group, although the difference was not statistically significant. However, there was a statistically significant decrease in the epithelium height of seminiferous tubules in the HS group (*p* = 0.015) (Table 1) (Figure 1(a)).

#### 3.1.3. Sperm Morphology and Vitality

**Figure 2:**
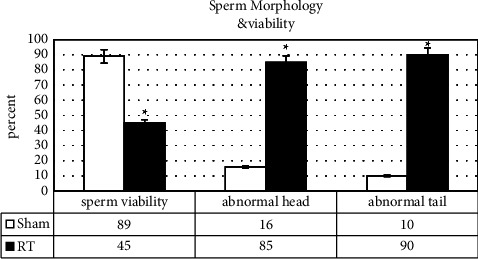
Evaluation of sperm morphology and viability following the induced heat stress. Sperm head and tail Morphology was evaluated in sham and HS groups. Sperm viability was reported as a percentage of the value in the sham and HS groups. Values are expressed as mean ± SD. *p* < 0.05 are significant. ^*∗*^*p* < 0.05 as compared with sham.

The results demonstrated that the mean percentage of sperm with normal morphology in the HS group decreased significantly compared with the sham group. In the HS group, only 15% of sperms had a normal head and 10% had a normal tail. 75% of the sperms were headless, and 50% had amputated tails. Also, the results of the eosin Y staining method showed that the induction of RT leads to a significant decrease in the viability of sperm in the HS group (*p* = 0.021) (Figures 1(b) and 2).

#### 3.1.4. Sperm Motility

**Figure 3:**
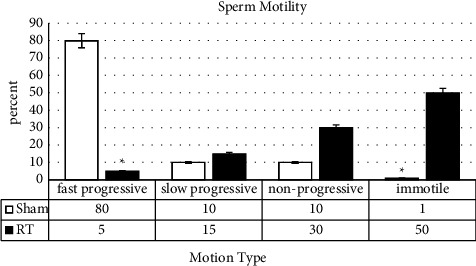
Evaluation of sperm motility following the induced heat stress. Four types of sperm motility were assessed for both sham and HS groups: fast progressive, slow progressive, nonprogressive, and immotile. Values are expressed as mean ± SD. *p* < 0.05 are significant. ^*∗*^*p* < 0.05 as compared with sham.

To compare the motility of adult sperms, four types of movement including rapid forward movement, slow forward movement, vibration (without moving forward), and no movement were assessed in both sham and HS groups. This test demonstrated that rapid forward motility decreased significantly in the HS group. Only 5% of the sperms in the HS group showed rapid forward movement, and 50% of the sperms in this group did not move at all (*p* = 0.012) (Figure 3).

### 3.2. Immunohistochemistry Test Results

#### 3.2.1. LC3*β* and Beclin-1 Proteins

**Figure 4:**
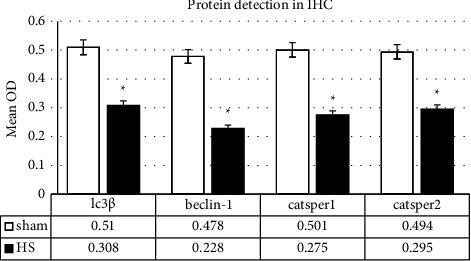
Evaluation of sperm autophagy marker (LC3*β*, Beclin-1) and CatSper proteins following the induced heat stress in testicles. The reported optical density of LC3*β*, Beclin-1, CatSper1, and CatSper2 proteins in sham and HS groups. Values are expressed as mean ± SD. *p* < 0.05 are significant. ^*∗*^*p* < 0.05 as compared with sham. OD: optical density.

A positive reaction for the expression of LC3*β* and Beclin-1 in the sham group indicates that sperm autophagy is active under normal conditions. In the HS group, the number of sperms and the distribution of these proteins in the sperm were reduced. According to the data analysis, in the seminiferous epithelium, a significant difference in the intensity of the reaction to anti-Beclin-1 and LC3*β* antibodies was observed between the sham and HS groups (*p* = 0.01) (Figures 1(a), 1(b) and 4).

#### 3.2.2. CatSper1 and CatSper2 Proteins

The results of the analysis on the intensity of the CatSper 1,2 reaction in sperm smears of the sham groups demonstrated that these proteins were largely located in the sperm's mid-tail. The intensity of immunohistochemical reaction for CatSper 1,2 proteins in the sperm's mid-tail was significantly reduced in the HS group compared with the sham group. Moreover, no statistically significant difference between the two groups was observed regarding the reaction intensity in the middle piece(*p* = 0.033) (Figures 1(a), 1(b) and 4).

### 3.3. Real-Time Test Result Analysis

**Figure 5:**
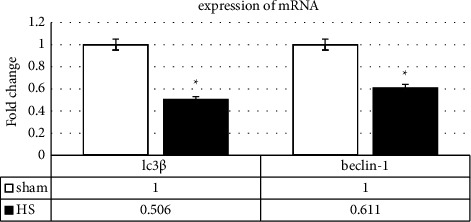
mRNA expression on Beclin-1 and LC3*β* following the induced heat stress in the sham and HS mice sperm as measured by the real-time PCR. Values are expressed as mean ± SD. *p* < 0.05 are significant. ^*∗*^*p* < 0.05 as compared with sham. The chart of Beclin-1 and MAP LC3*β* mRNA levels, as autophagy markers.

According to the results of real-time analysis, it was observed that the expression of autophagic index genes, including LC3*β* and Beclin-1, significantly increased in the HS group compared with the sham group (*p* = 0.005) (Figure 5).

## 4. Discussion

In the present study, by inducing heat stress, the activities of autophagy along with the change of CatSper calcium channels in the sperm of adult mice were evaluated.

The process of autophagy is a process recognized as a fundamental and essential factor for sperm formation in the development of the male gamete [4, 18]. Several studies have been conducted on the role of autophagy in spermiogenesis [19]. Nonetheless, conflicting reports have been published regarding the impact of calcium ions on the inhibition or induction of autophagy [4]. There are four proteins in the CatSper action channel subunit, of which CatSper1 and CatSper2 are essential for sperm hyperactivation and fertility [20, 21]. In the present study, through molecular and immunohistochemical techniques, the distribution and natural expression of CatSpers 1 and 2 along with the natural activity of autophagic indices including LC3 and Beclin-1 were identified in the sperms of nonintervention mice. It has been reported that calcium channels in tissues such as heart muscle and axon neurons are highly sensitive to heat stress and intracellular calcium homeostasis has changed dramatically following HS [7, 22]. Regarding the relationship between heat stress and calcium channels, in the present study, the RT model was used to induce heat stress in the testis. This study demonstrated that mice whose testes were in the suprascrotal position (RT) for 120 hours had severely impaired spermatogenesis and several abnormalities in sperm parameters, including motility, viability, and morphology. In this regard, several studies have indicated that heat stress causes testicular weight loss and abnormal sperm parameters, leads to a decrease in testosterone and LH, and enhances FSH by inducing retractile testes in mice [23]. It is also shown that HS can lead to impaired spermatogenesis by reducing the concentration of proteins associated with tight junctions (TJs) in the blood-testis barrier (BTB) [24]. It has also been suggested that the most substantial cause of impaired spermatogenesis is the ROS produced during HS [25]. Most studies have hypothesized the direct involvement of ROS due to heat stress as a cause of impaired sperm parameters.

In the present study, by stressing the role of temperature increase on CatSper1 and CatSper2 calcium channel dysfunction, we illustrated that heat stress might influence sperm parameters by impairing calcium homeostasis-dependent autophagy. The immunohistochemical results of the current study demonstrated a decrease in the distribution of CatSper1 and CatSper2 proteins in sperms after HS, which is accompanied by impaired spermatogenesis and sperm parameters, e.g., sperm morphology and motility. These results indicate the sensitivity of these channels after 120 hours of exposure to body temperature.

Studies have implied that part of the CatSper1 complex contains an HSP70 protein (heat shock protein) to prevent protein denaturation during HS [26]. But it seems in this study that due to the sharp decrease in CatSper1, this protein could not protect these calcium channels against heat stress. In the present study, after 120 hours of exposure to body temperature, decreased expression and distribution of CatSper1 and CatSper2 along with reduced whip-like movements of sperm indicate impaired calcium homeostasis. Possibly, calcium could not enter the mitochondria and provide the ATP required for hyperactivity. However, other studies have suggested that sperm hyperactivity is the result of the physiological activity of autophagy-related proteins, including LC3 and Beclin. So, by inhibiting the mentioned proteins, sperm motility was severely reduced [27]. Also, the results of the real-time technique in the present study demonstrated induction of HS along with a significant decrease in autophagic markers including LC3 and Beclin in sperms. Furthermore, immunohistochemical results of spermatogenesis of tubules at stages 7 and 8 on testicular tissue demonstrated a decline in the distribution of autophagic markers including LC3 and Beclin in sperms. Autophagy is incredibly active in converting spermatids to sperm and breaking off cytoplasmic bridges to release sperm at stages 7 and 8 of spermatogenesis. In the present study, a decrease in Johnson's score at stages 7 and 8 and a decrease in the expression of LC3 and Beclin proteins suggest unsuccessful autophagic activity. Other researchers have reported an increase in autophagy after HS which is inconsistent with our results. They have stated that in the sperm sample of adults with a history of cryptorchidism in the prepubertal period, mitophagy occurs due to the removal of mitochondria through autophagy as a result of decreased motility and ATP in adult sperms, which is inconsistent with the results of our study [28]. This inconsistency might be due to the different impacts of HS induction on the testis in the prepubertal and postpubertal periods since HS was induced during puberty in the current study. In this study, following heat stress, the decrease in CatSper protein distribution may lead to the structural disorder of CatSper channels, which could strongly affect autophagic activity. Also, disruption of spermatogenesis and sperm parameters may be the consequence of decreased autophagy activity. In this study, calcium concentration following damage to CatSper1 and CatSper2 was not measured. It is suggested that future research studies evaluate the autophagic calcium-mediated molecular pathway following HS.

## Figures and Tables

**Table 1:** Comparison of the seminiferous histological marker in sham and HS groups.

	Sham	HS
Modified Johnsenscore	9.54	3.45^*∗*^^(0.022)^
Diameter, *µ*m (mean ± SD)	255 ± 3.7	193.07 ± 22.63
Epithelium thickness, *µ*m (mean ± SD)	70 ± 3.57	31.71 ± 7.37^*∗*^^(0.015)^

The table shows the evaluation of testicular modified Johnsen score, seminiferous tubule diameter, and epithelium height following the induced heat stress. Values are expressed as mean ± SD. *p* < 0.05 are significant; ^*∗*^*p* < 0.05 vs. sham.

## Data Availability

The data used to support the findings of this study are available upon request from the corresponding author. The data are not publicly available due to privacy or ethical restrictions.
